# Selective inhibition of intestinal guanosine 3′,5′-cyclic monophosphate signaling by small-molecule protein kinase inhibitors

**DOI:** 10.1074/jbc.RA118.002835

**Published:** 2018-04-13

**Authors:** Marcel J. C. Bijvelds, Gary Tresadern, Ann Hellemans, Karine Smans, Natascha D. A. Nieuwenhuijze, Kelly F. Meijsen, Jean-Pierre Bongartz, Luc Ver Donck, Hugo R. de Jonge, Jan A. J. Schuurkes, Joris H. De Maeyer

**Affiliations:** From the ‡ Department of Gastroenterology and Hepatology, Erasmus MC University Medical Center, P. O. Box 2040, 3000CA Rotterdam, The Netherlands,; § Janssen Research and Development, a Division of Janssen Pharmaceutica NV, Turnhoutseweg 30, B-2340 Beerse, Belgium, and; ¶ Shire-Movetis NV, Veedijk 58, B-2300 Turnhout, Belgium

**Keywords:** protein kinase G, PKG, drug discovery, signal transduction, intestinal epithelium, cyclic GMP, cGMP, CFTR, cystic fibrosis transmembrane conductance regulator, enterotoxigenic E. coli, ETEC, secretory diarrhea, vasodilator-stimulated phosphoprotein

## Abstract

The guanosine 3′,5′-cyclic monophosphate (cGMP)-dependent protein kinase II (cGKII) serine/threonine kinase relays signaling through guanylyl cyclase C (GCC) to control intestinal fluid homeostasis. Here, we report the discovery of small-molecule inhibitors of cGKII. These inhibitors were imidazole-aminopyrimidines, which blocked recombinant human cGKII at submicromolar concentrations but exhibited comparatively little activity toward the phylogenetically related protein kinases cGKI and cAMP-dependent protein kinase (PKA). Whereas aminopyrimidyl motifs are common in protein kinase inhibitors, molecular modeling of these imidazole-aminopyrimidines in the ATP-binding pocket of cGKII indicated an unconventional binding mode that directs their amine substituent into a narrow pocket delineated by hydrophobic residues of the hinge and the αC-helix. Crucially, this set of residues included the Leu-530 gatekeeper, which is not conserved in cGKI and PKA. In intestinal organoids, these compounds blocked cGKII-dependent phosphorylation of the vasodilator-stimulated phosphoprotein (VASP). In mouse small intestinal tissue, cGKII inhibition significantly attenuated the anion secretory response provoked by the GCC-activating bacterial heat-stable toxin (STa), a frequent cause of infectious secretory diarrhea. In contrast, both PKA-dependent VASP phosphorylation and intestinal anion secretion were unaffected by treatment with these compounds, whereas experiments with T84 cells indicated that they weakly inhibit the activity of cAMP-hydrolyzing phosphodiesterases. As these protein kinase inhibitors are the first to display selective inhibition of cGKII, they may expedite research on cGMP signaling and may aid future development of therapeutics for managing diarrheal disease and other pathogenic syndromes that involve cGKII.

## Introduction

The guanosine 3′,5′-cyclic monophosphate (cGMP)-dependent protein kinase II (cGKII)^2^ serine/threonine kinase was originally identified and characterized as a major cGMP target in brush border membranes originating from rat and pig intestinal epithelium and was subsequently cloned from rat brain and intestinal tissue (1–4). Like cGKI, the only other cGMP-dependent protein kinase identified in vertebrates, cGKII consists of an N-terminal regulatory domain followed by two cGMP-binding domains arranged in tandem and a C-terminal catalytic domain (5). The N-terminal domain contains a leucine zipper motif that enables dimerization and carries a myristoyl moiety that links cGKII to lipid membranes. It is thought that cGMP binding, through a conformational change, relieves steric inhibition by a pseudosubstrate domain, located just downstream of the leucine zipper motif, rendering the enzyme active (6). Although cGKII forms a dimeric structure, there is no evidence for cooperative substrate (Mg^2+^-ATP) binding, and, based on the close homology to the catalytic domain of PKA, each monomer is projected to form a fully functional catalytic unit. However, because the monomers are thought to be arranged in parallel, dimerization may stabilize membrane anchoring via the paired myristoyl moieties. Plasma membrane anchoring has been shown to be essential for the activation of one of its key substrates in intestinal tissue, the cystic fibrosis transmembrane conductance regulator (CFTR) anion channel (7).

The gene encoding cGKII (*PRKG2*) is abundantly expressed in intestinal epithelium and to a more limited extent in various other tissues of both epithelial and nonepithelial origin. In intestinal epithelium, cGKII relays signaling through a membrane-associated, cGMP-producing enzyme, guanylyl cyclase C (GCC). The catalytic activity of this receptor-enzyme is triggered by two locally produced ligands, the peptides guanylin and uroguanylin, but also by the heat-stable toxin (STa) produced by enterotoxigenic *Escherichia coli* (ETEC) strains. The (uro)guanylin/GCC/cGMP signaling axis stimulates intestinal salt and water secretion through coordinate activation of CFTR-dependent chloride and bicarbonate secretion and inhibition of sodium uptake through sodium-proton exchanger isotype 3 (NHE3) (8). Dysregulation of this pathway may lead to luminal dehydration and intestinal obstruction as well as secretory diarrhea (8–10). Indeed, ETEC-provoked secretory diarrhea is a significant cause of mortality in young children (11).

In addition to its role in intestinal fluid homeostasis, one of the principal physiological roles of cGKII appears to be the regulation of the cell cycle and cellular differentiation in specific tissues. Thus, the most prominent phenotype of cGKII deficiency in rodents (and cattle) is dwarfism, which is caused by a defect in endochondral ossification, resulting from an impaired hypertrophic differentiation of chondrocytes (12–15).

Apart from the intestinal epithelium and growth plate cartilage, cGKII is found in various regions of the brain with relatively high expression in specific nuclei (16). cGKII appears to modulate synaptic transmission, and *Prkg2*-null mice display subtle learning, emotional, and behavioral deficiencies (17, 18).

cGMP has several effectors other than cGKI and cGKII, including PKA (by direct cross-activation), several phosphodiesterases (PDEs), and cyclic nucleotide–gated cation channels. Therefore, to establish the cGKI or cGKII dependence of cGMP-dependent signaling events, blockers of the cGMP-dependent protein kinases, which either antagonize binding of cGMP or peptide substrate, have been developed (19, 20). However, the application of these (*R*_p_)-cGMP isomers (carrying a sulfur substitution on the phosphate group) and inhibitor peptides, respectively, is for the most part limited to *in vitro* studies (19). The compound KT5823 (structurally related to the broad-specificity protein kinase inhibitor staurosporine) has been used as a blocker of cGMP-dependent protein kinases, but its efficacy and selectivity have been questioned (20, 21). Moreover, these inhibitors cannot readily be used to discern between cGKI- and cGKII-mediated effects. Here, we report the discovery of a set of imidazole-aminopyrimidines that inhibit cGKII activity *in vitro* and in native intestinal tissue.

## Results

### Selection of compounds

**Figure 1.**
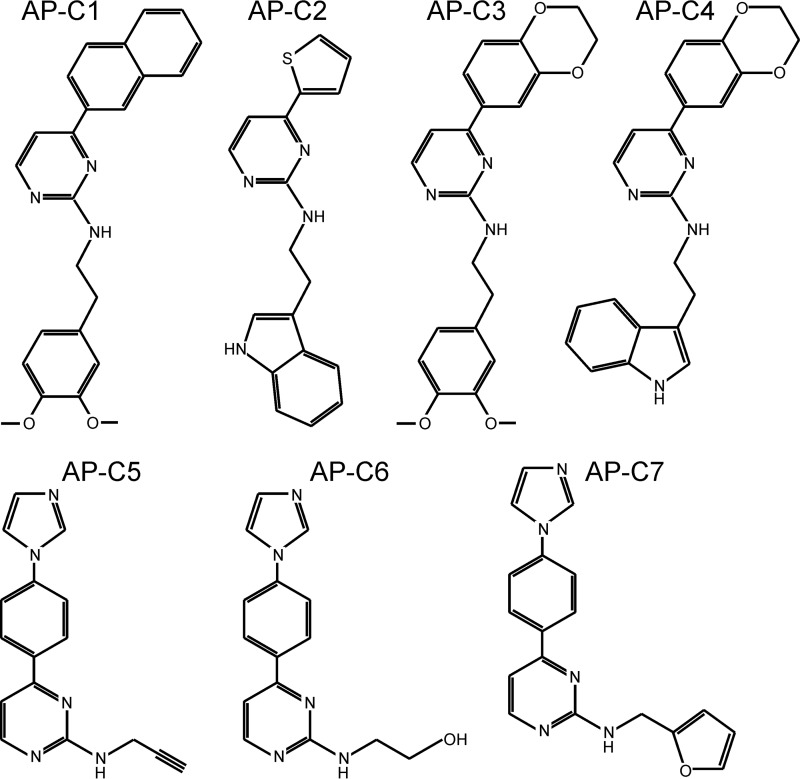
**Chemical structure of tested aminopyrimidines.**

A panel of aminopyrimidines (Fig. 1) that were shown to inhibit cGMP-dependent protein phosphorylation by recombinant human cGKII by >50% at 10 μmol/liter (Table 1) were tested for their ability to inhibit cGKII in intact tissue/cells by assessing their effect on cGMP-induced anion secretion in mouse ileum (*ex vivo*). It has been shown previously that the short-circuit current (Isc) response of mouse ileum to 8-pCPT-cGMP fully depends on cGKII and the CFTR anion channel (22). This test identified two compounds, AP-C5 and AP-C6, which showed 1) potent inhibition of cGMP-dependent cGKII-mediated protein phosphorylation and 2) effective inhibition of cGMP-dependent, CFTR-mediated anion secretion in intestinal tissue (Table 1). In contrast, compounds AP-C1 and AP-C3, although relatively potent inhibitors of cGKII *in vitro*, only weakly inhibited cGKII-dependent anion secretion, suggesting that these compounds do not effectively partition intracellularly or, alternatively, are actively secreted by intestinal cells.

**Table 1:** **Identification of compounds that inhibit cGKII activity in intestinal tissue** For assessing pharmacological inhibition of intestinal cGKII-dependent anion secretion, compounds were selected with pIC_50_ (−log_10_ of the half-maximal inhibitory concentration) ≥5 as assessed in assays of recombinant human cGKII-mediated protein phosphorylation. Their effect on cGKII-dependent anion secretion was assessed on mouse ileum in an Ussing chamber setup. Inhibition of 8-pCPT-cGMP (50 μmol/liter)–dependent anion secretion was calculated as the residual Isc response 20 min after addition of compound (20 μmol/liter) relative to the initial full Isc response assessed just before addition of the compound. The number of biological replicates is indicated in parentheses.

Compound	pIC_50_ *in vitro*	8-pCPT-cGMP–dependent anion secretion (% inhibition)
AP-C1	6.5	1.7 ± 10.8 (3)
AP-C2	5.2	−7.7 ± 16.7 (3)
AP-C3	6.3	7.6 ± 10.0 (4)
AP-C4	5.2	−3.7 ± 5.2 (3)
AP-C5	7.2	72.0 ± 7.0 (4)
AP-C6	6.5	34.0 ± 12.7 (4)
AP-C7	5.0	5.5 ± 5.6 (4)

### In vitro protein kinase inhibition

**Figure 2.**
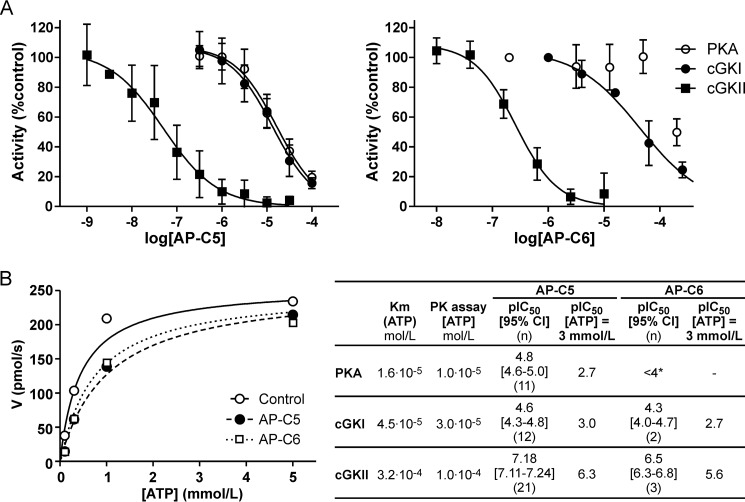
**Inhibition of PKA, cGKI, and cGKII by compounds AP-C5 and AP-C6.** *A*, phosphorylation of peptide substrate by PKA, cGKI, and cGKII was assessed at compound concentrations ranging between 1.0·10^−9^ and 3.0·10^−4^ mol/liter. The concentration dependence of enzyme inhibition was analyzed, yielding the pIC_50_ values shown in the *inset*. The number of technical replicates (*n*) is indicated in *parentheses*. Combined with the *K_m_*_(ATP)_ values determined in a separate set of experiments, these were used to estimate the pIC_50_ values at cellular ATP levels (44). *B*, rate of cGKII-dependent ^32^P_i_-peptide production (*V*) as a function of the ATP concentration in the absence (control) or presence of AP-C5 (0.1 μmol/liter) or AP-C6 (0.5 μmol/liter). Data were derived from a single experiment performed in triplicate. *, no complete inhibition was attained in the concentration range tested. *Error bars* represent S.D.

Compounds AP-C5 and AP-C6 concentration-dependently inhibited human cGKII activity *in vitro*. For both compounds, half-maximal inhibition was attained at submicromolar levels (Fig. 2*A*). To evaluate the selectivity of these two compounds, we also assessed their activity toward cGKI and PKA, *i.e.* protein kinases that are phylogenetically and structurally closely related to cGKII. This showed that the potency of inhibition of cGKI and PKA was markedly lower than of cGKII (Fig. 2*A*).

Most small-molecule inhibitors of protein kinases target the ATP-binding pocket (21). Consistent with this notion, we observed that the level of cGKII inhibition caused by AP-C5 or AP-C6 depended on the ATP concentration (Fig. 2*B*). These results suggest that these compounds compete with substrate for binding to the catalytic domain of cGKII. Interestingly, the reported *K_m_*_(ATP)_ values of cGKI and PKA are approximately 10-fold lower than that of cGKII (23, 24). Assuming that these related enzymes are also inhibited through an ATP-competitive mechanism, this suggests that these compounds would be highly cGKII-selective at cellular ATP levels.

### Molecular modeling of ligand docking in the ATP-binding pocket

**Figure 3.**
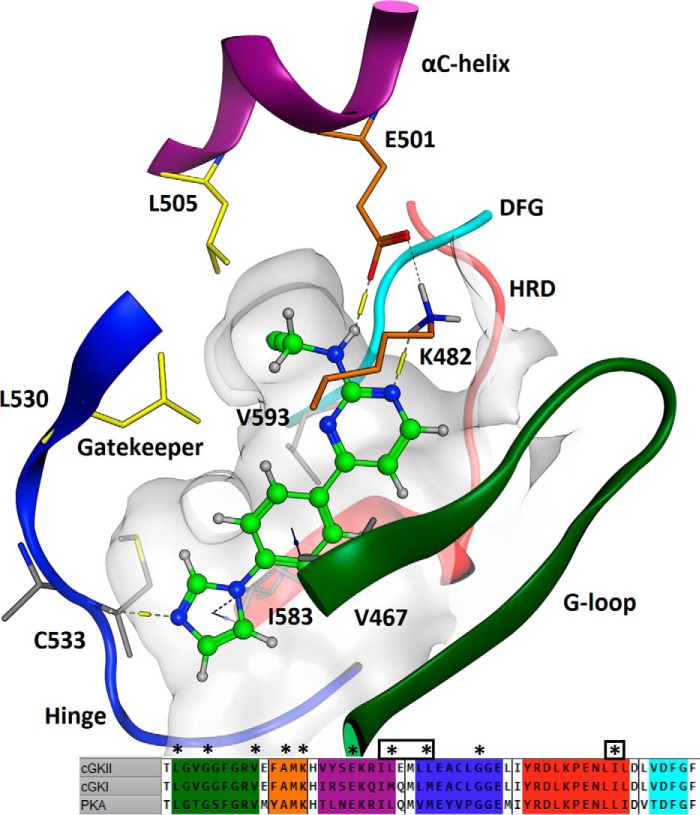
**Schematic of the docking of AP-C5 at the ATP-binding domain of cGKII.** This model was based on the X-ray structure of the highly homologous catalytic subunit of PKA. The numbered amino acids designate residues involved in ligand docking. Crucial structural elements of the active site are highlighted as follows: G-loop, *green*; Hyd1 (A*X*K β3-strand) residues, *orange*; αC-helix, *magenta*; hinge, *blue*; HRD motif, *red*; DFG motif, *turquoise*. The *inset* shows an alignment of the ATP-binding pockets of cGKI, cGKII, and PKA. *, residues that are within a 4.5-Å radius of the ligand. A *boxed asterisk* indicates a residue that is not conserved in cGKI and/or PKA.

In the proposed ligand docking solution (Fig. 3), the three linear aromatic rings of AP-C5 are wedged between hydrophobic residues in the G-loop and similar residues flanking the DFG and HRD motifs, binding through hydrogen–π interactions with Val-467, Ile-583, and Val-593. The aminopyrimidine ring forms hydrogen bonds with catalytic residues Lys-482 and Glu-501, and the imidazole ring acts as a hydrogen-bond acceptor to the backbone NH group of Cys-533 in the hinge region. This binding mode directs the acetenyl amine substituent into a small pocket formed by Leu-505 in the αC-helix and the Leu-530 gatekeeper. Gatekeeper residues delimit the size of the ATP-binding pocket and are known to govern the affinity for substrates and inhibitors/modulators of protein kinases (21, 25). In cGKII, the pocket formed by Leu-505 and Leu-530 can accommodate acetenyl (AP-C5) or hydroxyl (AP-C6) substituents but not the larger furanyl moiety of AP-C7, accounting for the modest activity of the latter compound. In addition, because this pocket is lined with further hydrophobic residues (Val-514, Val-593, and Phe-595), the acetenyl group of AP-C5 is preferred above the more polar alcohol of AP-C6.

### Inhibition of intestinal cGKII

**Figure 4.**
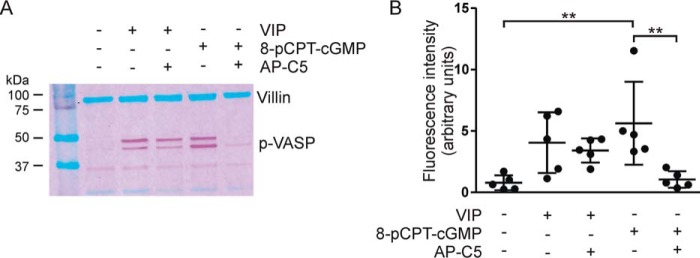
**cGKII-, but not PKA-, dependent VASP phosphorylation in intestinal organoids is blocked by AP-C5.** VASP phosphorylation at Ser-239 was detected by immunoblotting in mouse intestinal organoids. *A*, effect of AP-C5 on 8-pCPT-cGMP– and VIP-dependent VASP phosphorylation (*p-VASP*). Note that phosphorylation of Ser-157, a PKA-preferred residue, leads to a shift in the electrophoretic mobility of VASP (*upper* band of the doublet). *Numerals* to the *left* of the blot refer to the molecular mass (kDa) of protein standards shown in the *left outer lane. B*, aggregate data depicting the fluorescence intensity of the VASP signal relative to the villin signal of the same sample. Each data point represents one technical replicate. **, *p* < 0.01. *Error bars* represent S.D.

The vasodilator-stimulated phosphoprotein (VASP), through its association with actin filaments, plays an important role in cytoskeletal dynamics (26). Like cGKII and CFTR, VASP is located at the apical aspect of intestinal epithelial cells, and it has been shown that VASP is a substrate of cGMP-dependent protein kinases (27, 28). We found that incubation of ileal organoid cultures with 8-pCPT-cGMP markedly enhanced phosphorylation of VASP at Ser-239 (conforming to the topology of human VASP; the equivalent residue in murine VASP is actually at position 235), which is the site preferentially phosphorylated by cGMP-dependent protein kinases (Fig. 4) (27). This 8-pCPT-cGMP–dependent VASP phosphorylation was blocked by AP-C5, attesting the action of this compound on cellular cGKII. Consistent with its low activity toward PKA *in vitro*, AP-C5 did not block vasoactive intestinal peptide (VIP)-stimulated, PKA-mediated Ser-239 phosphorylation (Fig. 4).

**Figure 5.**
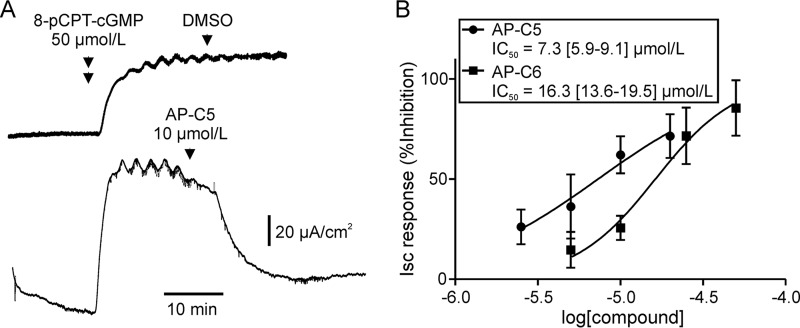
**Concentration-dependent inhibition of cGKII-dependent anion secretion in mouse ileum by AP-C5 and AP-C6.** *A*, representative Ussing chamber experiment illustrating the procedure for testing inhibition of cGKII-dependent, CFTR-mediated anion secretion. Anion secretion was stimulated by addition of 8-pCPT-cGMP. After the ensuing Isc response had reached a plateau, compound or vehicle (DMSO) was added to the luminal bath. Inhibition of ileal anion secretion was calculated as the reduction in the Isc response 20 min after addition of compound relative to the initial full Isc response assessed just before addition of the compound. *B*, inhibition of 8-pCPT-cGMP–dependent anion secretion as a function of compound concentration. Data of six (AP-C5) or five (AP-C6) biological replicates were analyzed by nonlinear regression, yielding the IC_50_ values (mean with 95% confidence interval in *square brackets*) shown in the *inset. Error bars* represent S.D.

In mouse ileum, AP-C5 and AP-C6 concentration-dependently inhibited 8-pCPT-cGMP–induced anion secretion (Fig. 5). Half-maximal inhibition was attained at concentrations roughly 10 times higher than anticipated, based on the estimated pIC_50_ values for cGKII inhibition at cellular ATP levels (see Fig. 2, *inset*). This may indicate that, at high levels, cGKII activity does not limit anion secretion because saturating levels of substrate phosphorylation are attained. Furthermore, the activity of these substrates may not limit the rate of anion secretion. This holds true for intestinal CFTR as it has been shown that ≤50% of the cellular pool is required for a full-anion secretory response (29). Consequently, at the concentration where half-maximal inhibition of anion secretion occurs, the level of cGKII activity and substrate phosphorylation may be well below 50% of control values, and the level of cGKII inhibition attained may be in closer agreement with the data obtained *in vitro* than is immediately apparent.

**Figure 6.**
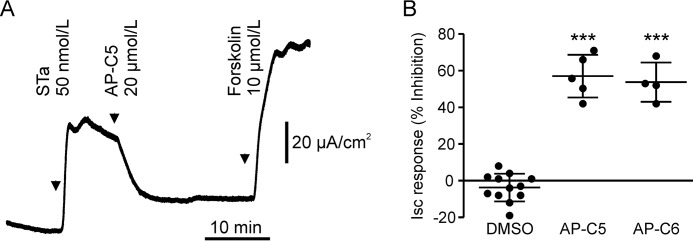
**Inhibition of cGKII by AP-C5 or AP-C6 reduces STa-dependent anion secretion in mouse ileum.** *A*, representative experiment illustrating the procedure for testing inhibition of STa-dependent anion secretion. Note that in the sustained presence of compound the tissue remained responsive to forskolin. *B*, both compounds (20 μmol/liter) reduced STa-dependent anion secretion relative to a solvent (0.1% DMSO) control. Each data point represents one biological replicate. ***, *p* < 0.001 *versus* DMSO. *Error bars* represent S.D.

The STa produced by ETEC provokes secretory diarrhea by potent activation of GCC (8). It has been shown that the anion secretory response elicited by STa is reduced by approximately 80% in cGKII-deficient mice compared with normal animals (22). Congruent with this partial cGKII dependence, we found that AP-C5 and AP-C6 partially blocked the STa-mediated Isc response in mouse ileum (Fig. 6).

### Effect of AP-C5 and AP-C6 on PDE activity

**Figure 7.**
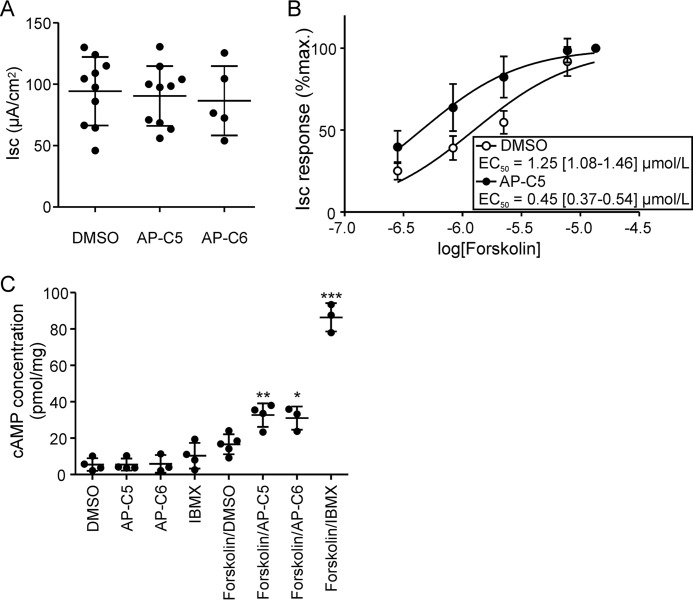
**AP-C5 and AP-C6 potentiate cAMP signaling by PDE inhibition.** *A*, in ileum, AP-C5 and AP-C6 did not reduce the Isc response elicited by forskolin (10 μmol/liter). Each data point represents one biological replicate. *B*, forskolin-dependent anion secretion in the presence of AP-C5 (50 μmol/liter) or vehicle (DMSO). AP-C5 lowered the forskolin concentration required to attain half-maximal stimulation of the Isc (*p* < 0.0001; *inset*). Data of 10 biological replicates per group were analyzed. *C*, compounds AP-C5 and AP-C6 potentiate forskolin-induced cAMP production in T84 cells. T84 cells were incubated with cGKII inhibitor compound (50 μmol/liter), DMSO (0.1%), or isobutylmethylxanthine (*IBMX*) (0.5 mmol/liter) in the absence or presence of forskolin (0.5 μmol/liter). Each data point represents one technical replicate. *, *p* < 0.05; **, *p* < 0.01; ***, *p* < 0.001 *versus* forskolin/DMSO. *Error bars* represent S.D.

In mouse ileum, we found that these compounds, at a concentration ≤50 μmol/liter, did not inhibit the anion secretory response elicited by the cAMP/PKA agonist forskolin (Fig. 7*A*). In fact, we found that AP-C5 potentiated the anion secretory response to forskolin of ileum (Fig. 7*B*). In view of this potentiation, we speculated that this compound, and its close analog AP-C6, might elevate cAMP levels, conceivably through inhibition of PDE activity. Consistent with this supposition, we found that in T84 cells these compounds moderately enhanced cAMP levels but only in the presence of forskolin (*i.e.* of adenylyl cyclase activity; Fig. 7*C*). This strongly suggests that these compounds, at high concentrations, potentiate cAMP signaling through inhibition of PDE activity.

## Discussion

We report the discovery of small-molecule inhibitors of cGKII that block protein phosphorylation and the ensuing ion secretory responses in the small intestinal epithelium elicited by cGMP-linked signal transduction pathways. *In vitro*, these imidazole-aminopyrimidines inhibited human cGKII at submicromolar levels. In contrast, these compounds showed little activity toward phylogenetically related ACG-type serine/threonine kinases (cGKI and PKA). To our knowledge, the presently developed compounds are the first to display selectivity in this respect and activity in native (intestinal) tissue.

Our kinetic analysis indicates that these compounds interact with the catalytic (ATP-binding) domain of cGKII. Aminopyrimidyl motifs are relatively common in this type of protein kinase inhibitors as these readily form hydrogen bonds with residues in the hinge region of the catalytic domain. However, molecular modeling indicated that the binding mode of the present set of compounds is more unusual as the aminopyrimidyl ring is projected to interact with residues in the catalytic loop. A similar, *reversed* orientation has previously been described for blockers of PDK1 and PIM1 (30, 31). This orientation guides the amine substituent into a small pocket, delimited by the Leu-505 and Leu-530 (gatekeeper) residues. These residues form a selectivity pocket that permits docking of imidazole-aminopyrimidines with relatively small substituents, such as AP-C5 and AP-C6. Importantly, both cGKI (Met-438) and PKA (Met-121) contain slightly bulkier gatekeeper residues, which preclude effective docking of the present set of ligands. In addition, in cGKI, another methionine residue (Met-413) replaces the Leu-505 in the αC-helix of cGKII, further restricting access to the selectivity pocket. Thus, the unusual binding mode of AP-C5 accounts for both its potency and selectivity. High affinity results from polar interactions with residues in both the hinge and catalytic loops and hydrophobic interactions across the imidazole-phenylpyrimidine core, whereas the positioning of the amine substituent into a tight pocket, which only accommodates small, preferentially hydrophobic groups, confers selectivity.

In mouse small intestine, these compounds effectively blocked 8-pCPT-cGMP–dependent VASP phosphorylation and anion secretion but only partially inhibited the STa-provoked anion secretory response. These data corroborate previous studies on cGKII-deficient mice showing that the response to STa is only approximately 80% dependent on cGKII and that, in addition, STa signals through cGKII-independent routes. Chiefly, cGMP is thought to also enhance PKA signaling through competitive inhibition of the cAMP-hydrolyzing PDE type 3 (PDE3), but direct cross-activation of PKA may also occur (22, 32). Importantly, in contrast to cGMP itself, imidazole ring–substituted cGMP analogs do not block PDE activity (nor are they readily hydrolyzed by PDEs) or activate PKA. Consequently, the (prosecretory) effects of 8-pCPT-cGMP in the intestine are fully cGKII-dependent and amenable to inhibition by our compounds.

Apart from by a redundancy in prosecretory cGMP-dependent signaling pathways, the moderate efficacy of these cGKII inhibitors toward the STa-dependent Isc response of ileum may be compounded by their weak inhibition of PDEs, which may derive from their structural resemblance to previously identified PDE blockers (33). Thus, at high concentrations, they would have opposing effects on the cGMP-mediated secretory response by concurrent inhibition of cGKII and PDE activity.

Suppression of CFTR phosphorylation may effectively reduce fluid secretion as was shown for direct pharmacological CFTR blockage and for GCC inhibition (34, 35). However, ETEC/STa–provoked secretory diarrhea not only results from anomalous CFTR activation but also from inhibition of NHE3 activity (*i.e.* an antiabsorptive action) (8). Like cGKII, NHE3 is highly expressed in the villus region of the intestinal epithelium and was shown to be inhibited in the presence of 8-pCPT-cGMP or STa (36). Consequently, we infer that, upon ETEC colonization of the gut, pharmacological cGKII blockage would stimulate salt and fluid absorption by restoring NHE3 activity.

In addition to its established role in controlling the intestinal fluid balance and the cell cycle, evidence accrued over the last decade points toward various other functions of cGKII in physiology. These often intriguing new prospects, such as cGKII's potential role in (feeding) behavior and psychosocial disorders, warrant further research (17, 18, 37, 38). We propose that the compounds presented here may help to substantiate and further delimit the role of cGKII in such processes.

## Experimental procedures

### Animals

Mice (FVB) were maintained in an environmentally controlled facility at the Erasmus MC, Rotterdam, The Netherlands. All experiments were performed on animals 12–26 weeks of age and were approved of by the Ethical Committee for Animal Experiments of the Erasmus MC.

### Protein kinase assays

For assays performed on recombinant full-length human cGKI (Promega) or cGKII (His-tagged; produced through the baculovirus insect cell expression system), enzyme (100 μg/liter) was added to assay solution containing Na-cGMP (50 μmol/liter), DTT (5 mmol/liter), Tris/HCl, pH 7.4 (20 mmol/liter), MgCl_2_ (5 mmol/liter), BSA (25 mg/liter), and peptide substrate 2A3 (RRKVSKQE; 25 mg/liter) (39). For assays on the PKA catalytic subunit (25 μg/liter; Promega), the peptide substrate kemptide (LRRASLG; 25 mg/liter) was used, and Na-cGMP and DTT were omitted from the assay solution. Test compounds were added from stocks prepared in DMSO, and their effect was evaluated relative to the appropriate vehicle control (2% DMSO). Reactions (1 h at 22 °C) were started by addition of [γ-^32^P]ATP (100 MBq/liter; total ATP concentration as indicated in Fig. 2) and quenched by adding EDTA (100 mmol/liter). Peptide substrate was collected on cellulose phosphate cation exchanger (MAPHN0B50, Millipore) prerinsed with 1% H_3_PO_4_. After washing five times with 1% H_3_PO_4_, the radioactivity collected on the filters was quantified by liquid scintillation counting. For data analysis, median values, derived from assays performed in triplicate, were used.

### Molecular modeling of ligand docking

Because the X-ray structure of the cGKII catalytic domain is unresolved, we used a high-resolution (1.6-Å) X-ray structure of the PKA catalytic domain (Protein Data Bank code 4WB5) as a structural template for modeling of ligand binding (40). The cGKII (amino acids 453–711; UniProt accession number Q13237) and PKA (UniProt accession number P17612) catalytic domains were aligned (showing 51% sequence identity), and a homology model of the cGKII catalytic domain was built using Molecular Operating Environment (MOE) (v2016.0802; Chemical Computing Group, Montreal, Canada). The resulting model had a low backbone root-mean-square deviation in the binding site, no stereochemical violations or structural clashes, and suitable backbone and side-chain dihedral angles. Modeling of the ligands in ADMET Predictor (v7; Simulations Plus, Lancaster, CA) showed that AP-C5 and AP-C6 are uncharged at neutral pH values (p*K_a_* ≤ 5.3). Induced fit docking of the ligand in the ATP-binding site, defined as all amino acids and those bonded to them within a 4.5-Å radius of the ligand, was performed using MOE.

### Ussing chamber assays

Collection of murine intestinal tissue and Ussing chamber assays were performed as described elsewhere (41). Tissue was incubated in modified Meyler solution (128 mmol/liter NaCl, 4.7 mmol/liter KCl, 1.3 mmol/liter CaCl_2_, 1.0 mmol/liter MgCl_2_, 0.3 mmol/liter Na_2_HPO_4_, 0.4 mmol/liter NaH_2_PO_4_, 20 mmol/liter NaHCO_3_, 10 mmol/liter HEPES), supplemented with glucose (10 mmol/liter; added solely to the serosal bathing solution) in 95% O_2_, 5% CO_2_, pH 7.3, at 37 °C. CFTR-dependent anion secretion was stimulated by addition of the adenylyl cyclase activator forskolin (Sigma-Aldrich), STa (added to the luminal bathing solution only; Bachem), or the membrane-permeable cGMP analog 8-pCPT-cGMP (Sigma-Aldrich). Data shown represent Isc measurements, obtained by clamping of the transepithelial potential difference at 0 mV.

### VASP serine 239 phosphorylation

Organoids derived from mouse ileum were cultured as described elsewhere (42). Organoids (5 days after seeding) were incubated in advanced DMEM/F-12 (Invitrogen) with VIP (50 nmol/liter; Bachem) or 8-pCPT-cGMP (50 μmol/liter) and in the presence or absence of compound AP-C5 (20 μmol/liter; Fig. 1) in 5% CO_2_, pH 7.3, at 37 °C. After 30 min, medium was aspirated, and organoids were collected in ice-cold PBS supplemented with NaF (1 mmol/liter) and Na_3_VO_4_ (1 mmol/liter). After centrifugation (300 × *g*, 5 min), pelleted organoids were lysed in NaCl (150 mmol/liter), Tris/HCl, pH 7.6 (25 mmol/liter), Triton X-100 (1%), sodium deoxycholate (1%), SDS (0.1%), NaF (5 mmol/liter), Na_3_VO_4_ (3 mmol/liter) supplemented with a protease inhibitor mixture (Roche Applied Science). Lysates were subjected to SDS-PAGE, and proteins were transferred to nitrocellulose membrane. Ser-239–phosphorylated VASP was detected by Western blot analysis (CST3114, Cell Signaling Technology). A fluorescent dye–labeled secondary antibody and the Odyssey IR imaging system (Application software 3, LI-COR Biosciences) were used for quantitation. Detection of villin (SC58897, Santa Cruz Biotechnology) served as a reference.

### Measurement of cellular cAMP levels

T84 cells were grown to 80% confluence in 12-well culture plates (43). Cells, washed twice in modified Meyler solution, were incubated in modified Meyler solution supplemented with glucose (10 mmol/liter) and AP-C5 or AP-C6 (Fig. 1) or the broad-specificity PDE inhibitor isobutylmethylxanthine (Sigma-Aldrich) in the absence or presence of forskolin for 10 min in 5% CO_2_ at 37 °C. Reactions were quenched by adding ice-cold ethanol (70%), and cAMP levels were assayed as described elsewhere (41).

### Data analysis

The concentration dependence of protein kinase inhibition, Isc responses, and enzyme kinetics were analyzed by nonlinear regression (GraphPad Prism 5.0, GraphPad Software). Derived EC_50_ and IC_50_ values are presented as mean and 95% confidence interval. The Cheng–Prusoff equation was used to estimate pIC_50_ values at cellular ATP levels (44). VASP phosphorylation, inhibition of STa-dependent anion secretion, and cAMP production in T84 cells were evaluated by one-way analysis of variance followed by Tukey's multiple comparisons test (GraphPad Prism 5.0). Data are presented as mean ± S.D.

## Author contributions

M. J. B., H. R. d. J., and J. H. D. M. conceptualization; M. J. B. and G. T. data curation; M. J. B. and G. T. formal analysis; M. J. B., G. T., N. D. N., K. F. M., and H. R. d. J. investigation; M. J. B. and G. T. writing-original draft; A. H., J. A. S., and J. H. D. M. supervision; A. H., J. A. S., and J. H. D. M. funding acquisition; A. H. project administration; K. S., J.-P. B., and L. V. D. resources; K. S., J.-P. B., L. V. D., H. R. d. J., J. A. S., and J. H. D. M. writing-review and editing.

## Footnotes

2 The abbreviations used are:

cGK: cGMP-dependent protein kinase
GCC: guanylyl cyclase C
VASP: vasodilator-stimulated phosphoprotein
STa: heat-stable toxin
CFTR: cystic fibrosis transmembrane conductance regulator
ETEC: enterotoxigenic *E. coli*
NHE3: sodium-proton exchanger isotype 3
PDE: phosphodiesterase
8-pCPT-cGMP: 8-(4-chlorophenylthio)guanosine 3′,5′-cyclic monophosphate
Isc: short-circuit current
VIP: vasoactive intestinal peptide.

## References

[1] de Jonge H. R. (1976) Cyclic nucleotide-dependent phosphorylation of intestinal epithelium proteins. Nature 262, 590–593 10.1038/262590a0 183134

[2] de Jonge H. R. (1981) Cyclic GMP-dependent protein kinase in intestinal brush borders. Adv. Cyclic Nucleotide Res. 14, 315–333 6269385

[3] Jarchau T., Häusler C., Markert T., Pöhler D., Vanderkerckhove J., De Jonge H. R., Lohmann S. M., and Walter U. (1994) Cloning, expression, and in situ localization of rat intestinal cGMP-dependent protein kinase II. Proc. Natl. Acad. Sci. U.S.A. 91, 9426–9430 10.1073/pnas.91.20.9426 7937783 PMC44825

[4] el-Husseini A. E., Bladen C., and Vincent S. R. (1995) Molecular characterization of a type II cyclic GMP-dependent protein kinase expressed in the rat brain. J. Neurochem. 64, 2814–2817 7760063 10.1046/j.1471-4159.1995.64062814.x

[5] Vaandrager A. B., Hogema B. M., and de Jonge H. R. (2005) Molecular properties and biological functions of cGMP-dependent protein kinase II. Front. Biosci. 10, 2150–2164 10.2741/1687 15970484

[6] Taylor M. K., Ahmed R., Begley M., and Uhler M. D. (2002) Autoinhibition and isoform-specific dominant negative inhibition of the type II cGMP-dependent protein kinase. J. Biol. Chem. 277, 37242–37253 10.1074/jbc.M202060200 12093798

[7] Vaandrager A. B., Smolenski A., Tilly B. C., Houtsmuller A. B., Ehlert E. M., Bot A. G., Edixhoven M., Boomaars W. E., Lohmann S. M., and de Jonge H. R. (1998) Membrane targeting of cGMP-dependent protein kinase is required for cystic fibrosis transmembrane conductance regulator Cl^−^ channel activation. Proc. Natl. Acad. Sci. U.S.A. 95, 1466–1471 10.1073/pnas.95.4.1466 9465038 PMC19047

[8] Field M. (2003) Intestinal ion transport and the pathophysiology of diarrhea. J. Clin. Investig. 111, 931–943 10.1172/JCI200318326 12671039 PMC152597

[9] Romi H., Cohen I., Landau D., Alkrinawi S., Yerushalmi B., Hershkovitz R., Newman-Heiman N., Cutting G. R., Ofir R., Sivan S., and Birk O. S. (2012) Meconium ileus caused by mutations in GUCY2C, encoding the CFTR-activating guanylate cyclase 2C. Am. J. Hum. Genet. 90, 893–899 10.1016/j.ajhg.2012.03.022 22521417 PMC3376486

[10] Fiskerstrand T., Arshad N., Haukanes B. I., Tronstad R. R., Pham K. D., Johansson S., Håvik B., Tønder S. L., Levy S. E., Brackman D., Boman H., Biswas K. H., Apold J., Hovdenak N., Visweswariah S. S., et al (2012) Familial diarrhea syndrome caused by an activating GUCY2C mutation. N. Engl. J. Med. 366, 1586–1595 10.1056/NEJMoa1110132 22436048

[11] Lanata C. F., Fischer-Walker C. L., Olascoaga A. C., Torres C. X., Aryee M. J., Black R. E., Child Health Epidemiology Reference Group of the World Health Organization, and UNICEF (2013) Global causes of diarrheal disease mortality in children <5 years of age: a systematic review. PLoS One 8, e72788 10.1371/journal.pone.0072788 24023773 PMC3762858

[12] Tsuchida A., Yokoi N., Namae M., Fuse M., Masuyama T., Sasaki M., Kawazu S., and Komeda K. (2008) Phenotypic characterization of the Komeda miniature rat Ishikawa, an animal model of dwarfism caused by a mutation in Prkg2. Comp. Med. 58, 560–567 19149413 PMC2710756

[13] Chikuda H., Kugimiya F., Hoshi K., Ikeda T., Ogasawara T., Shimoaka T., Kawano H., Kamekura S., Tsuchida A., Yokoi N., Nakamura K., Komeda K., Chung U. I., and Kawaguchi H. (2004) Cyclic GMP-dependent protein kinase II is a molecular switch from proliferation to hypertrophic differentiation of chondrocytes. Genes Dev. 18, 2418–2429 10.1101/gad.1224204 15466490 PMC522991

[14] Pfeifer A., Aszódi A., Seidler U., Ruth P., Hofmann F., and Fässler R. (1996) Intestinal secretory defects and dwarfism in mice lacking cGMP-dependent protein kinase II. Science 274, 2082–2086 10.1126/science.274.5295.2082 8953039

[15] Koltes J. E., Mishra B. P., Kumar D., Kataria R. S., Totir L. R., Fernando R. L., Cobbold R., Steffen D., Coppieters W., Georges M., and Reecy J. M. (2009) A nonsense mutation in cGMP-depandant type II protein kinase (PRKG2) causes dwarfism in American Angus cattle. Proc. Natl. Acad. Sci. U.S.A. 106, 19250–19255 10.1073/pnas.0904513106 19887637 PMC2780805

[16] Hofmann F., Feil R., Kleppisch T., and Schlossmann J. (2006) Function of cGMP-dependent protein kinases as revealed by gene deletion. Physiol. Rev. 86, 1–23 10.1152/physrev.00015.2005 16371594

[17] Werner C., Raivich G., Cowen M., Strekalova T., Sillaber I., Buters J. T., Spanagel R., and Hofmann F. (2004) Importance of NO/cGMP signalling via cGMP-dependent protein kinase II for controlling emotionality and neurobehavioural effects of alcohol. Eur. J. Neurosci. 20, 3498–3506 10.1111/j.1460-9568.2004.03793.x 15610182

[18] Wincott C. M., Abera S., Vunck S. A., Tirko N., Choi Y., Titcombe R. F., Antoine S. O., Tukey D. S., DeVito L. M., Hofmann F., Hoeffer C. A., and Ziff E. B. (2014) cGMP-dependent protein kinase type II knockout mice exhibit working memory impairments, decreased repetitive behavior, and increased anxiety-like traits. Neurobiol. Learn. Mem. 114, 32–39 10.1016/j.nlm.2014.04.007 24752151 PMC4451455

[19] Gambaryan S., Butt E., Kobsar A., Geiger J., Rukoyatkina N., Parnova R., Nikolaev V. O., and Walter U. (2012) The oligopeptide DT-2 is a specific PKG I inhibitor only *in vitro*, not in living cells. Br. J. Pharmacol. 167, 826–838 10.1111/j.1476-5381.2012.02044.x 22612416 PMC3575782

[20] Burkhardt M., Glazova M., Gambaryan S., Vollkommer T., Butt E., Bader B., Heermeier K., Lincoln T. M., Walter U., and Palmetshofer A. (2000) KT5823 inhibits cGMP-dependent protein kinase activity *in vitro* but not in intact human platelets and rat mesangial cells. J. Biol. Chem. 275, 33536–33541 10.1074/jbc.M005670200 10922374

[21] Bain J., McLauchlan H., Elliott M., and Cohen P. (2003) The specificities of protein kinase inhibitors: an update. Biochem. J. 371, 199–204 10.1042/bj20021535 12534346 PMC1223271

[22] Vaandrager A. B., Bot A. G., Ruth P., Pfeifer A., Hofmann F., and De Jonge H. R. (2000) Differential role of cyclic GMP-dependent protein kinase II in ion transport in murine small intestine and colon. Gastroenterology 118, 108–114 10.1016/S0016-5085(00)70419-7 10611159

[23] Slice L. W., and Taylor S. S. (1989) Expression of the catalytic subunit of cAMP-dependent protein kinase in *Escherichia coli*. J. Biol. Chem. 264, 20940–20946 2687267

[24] Vaandrager A. B., Edixhoven M., Bot A. G., Kroos M. A., Jarchau T., Lohmann S., Genieser H. G., and de Jonge H. R. (1997) Endogenous type II cGMP-dependent protein kinase exists as a dimer in membranes and can be functionally distinguished from the type I isoforms. J. Biol. Chem. 272, 11816–11823 10.1074/jbc.272.18.11816 9115239

[25] Zhang C., Kenski D. M., Paulson J. L., Bonshtien A., Sessa G., Cross J. V., Templeton D. J., and Shokat K. M. (2005) A second-site suppressor strategy for chemical genetic analysis of diverse protein kinases. Nat. Methods 2, 435–441 10.1038/nmeth764 15908922

[26] Benz P. M., Blume C., Seifert S., Wilhelm S., Waschke J., Schuh K., Gertler F., Münzel T., and Renné T. (2009) Differential VASP phosphorylation controls remodeling of the actin cytoskeleton. J. Cell Sci. 122, 3954–3965 10.1242/jcs.044537 19825941 PMC2773194

[27] Lohmann S. M., and Walter U. (2005) Tracking functions of cGMP-dependent protein kinases (cGK). Front. Biosci. 10, 1313–1328 10.2741/1621 15769627

[28] Lawrence D. W., Comerford K. M., and Colgan S. P. (2002) Role of VASP in reestablishment of epithelial tight junction assembly after Ca^2+^ switch. Am. J. Physiol. Cell Physiol. 282, C1235–C1245 10.1152/ajpcell.00288.2001 11997237

[29] Högenauer C., Santa Ana C. A., Porter J. L., Millard M., Gelfand A., Rosenblatt R. L., Prestidge C. B., and Fordtran J. S. (2000) Active intestinal chloride secretion in human carriers of cystic fibrosis mutations: an evaluation of the hypothesis that heterozygotes have subnormal active intestinal chloride secretion. Am. J. Hum. Genet. 67, 1422–1427 10.1086/316911 11055897 PMC1287919

[30] Pierce A. C., Jacobs M., and Stuver-Moody C. (2008) Docking study yields four novel inhibitors of the protooncogene Pim-1 kinase. J. Med. Chem. 51, 1972–1975 10.1021/jm701248t 18290603

[31] Medina J. R., Becker C. J., Blackledge C. W., Duquenne C., Feng Y., Grant S. W., Heerding D., Li W. H., Miller W. H., Romeril S. P., Scherzer D., Shu A., Bobko M. A., Chadderton A. R., Dumble M., et al (2011) Structure-based design of potent and selective 3-phosphoinositide-dependent kinase-1 (PDK1) inhibitors. J. Med. Chem. 54, 1871–1895 10.1021/jm101527u 21341675

[32] Forte L. R., Thorne P. K., Eber S. L., Krause W. J., Freeman R. H., Francis S. H., and Corbin J. D. (1992) Stimulation of intestinal Cl^−^ transport by heat-stable enterotoxin: activation of cAMP-dependent protein kinase by cGMP. Am. J. Physiol. Cell Physiol. 263, C607–C615 10.1152/ajpcell.1992.263.3.C607 1329520

[33] Kauffman R. F., Schenck K. W., Utterback B. G., Crowe V. G., and Cohen M. L. (1987) *In vitro* vascular relaxation by new inotropic agents: relationship to phosphodiesterase inhibition and cyclic nucleotides. J. Pharmacol. Exp. Ther. 242, 864–872 2821228

[34] Thiagarajah J. R., Broadbent T., Hsieh E., and Verkman A. S. (2004) Prevention of toxin-induced intestinal ion and fluid secretion by a small-molecule CFTR inhibitor. Gastroenterology 126, 511–519 10.1053/j.gastro.2003.11.005 14762788

[35] Bijvelds M. J., Loos M., Bronsveld I., Hellemans A., Bongartz J. P., Ver Donck L., Cox E., de Jonge H. R., Schuurkes J. A., and De Maeyer J. H. (2015) Inhibition of heat-stable toxin-induced intestinal salt and water secretion by a novel class of guanylyl cyclase C inhibitors. J. Infect. Dis. 212, 1806–1815 10.1093/infdis/jiv300 25999056

[36] Foulke-Abel J., In J., Yin J., Zachos N. C., Kovbasnjuk O., Estes M. K., de Jonge H. R., and Donowitz M. (2016) Human enteroids as a model of upper small intestinal ion transport physiology and pathophysiology. Gastroenterology 150, 638–649.e8 10.1053/j.gastro.2015.11.047 26677983 PMC4766025

[37] Valentino M. A., Lin J. E., Snook A. E., Li P., Kim G. W., Marszalowicz G., Magee M. S., Hyslop T., Schulz S., and Waldman S. A. (2011) A uroguanylin-GUCY2C endocrine axis regulates feeding in mice. J. Clin. Investig. 121, 3578–3588 10.1172/JCI57925 21865642 PMC3223926

[38] Gong R., Ding C., Hu J., Lu Y., Liu F., Mann E., Xu F., Cohen M. B., and Luo M. (2011) Role for the membrane receptor guanylyl cyclase-C in attention deficiency and hyperactive behavior. Science 333, 1642–1646 10.1126/science.1207675 21835979

[39] Pöhler D., Butt E., Meissner J., Müller S., Lohse M., Walter U., Lohmann S. M., and Jarchau T. (1995) Expression, purification, and characterization of the cGMP-dependent protein kinases Iβ and II using the baculovirus system. FEBS Lett. 374, 419–425 10.1016/0014-5793(95)01168-E 7589584

[40] Cheung J., Ginter C., Cassidy M., Franklin M. C., Rudolph M. J., Robine N., Darnell R. B., and Hendrickson W. A. (2015) Structural insights into mis-regulation of protein kinase A in human tumors. Proc. Natl. Acad. Sci. U.S.A. 112, 1374–1379 10.1073/pnas.1424206112 25605907 PMC4321305

[41] Bijvelds M. J., Bot A. G., Escher J. C., and De Jonge H. R. (2009) Activation of intestinal Cl^−^ secretion by lubiprostone requires the cystic fibrosis transmembrane conductance regulator. Gastroenterology 137, 976–985 10.1053/j.gastro.2009.05.037 19454284

[42] Dekkers J. F., Wiegerinck C. L., de Jonge H. R., Bronsveld I., Janssens H. M., de Winter-de Groot K. M., Brandsma A. M., de Jong N. W., Bijvelds M. J., Scholte B. J., Nieuwenhuis E. E., van der Brink S., Clevers H., van der Ent C. K., Middendorp S., et al (2013) A functional CFTR assay using primary cystic fibrosis intestinal organoids. Nat. Med. 19, 939–945 10.1038/nm.3201 23727931

[43] Dharmsathaphorn K., McRoberts J. A., Mandel K. G., Tisdale L. D., and Masui H. (1984) A human colonic tumor cell line that maintains vectorial electrolyte transport. *Am. J. Physiol*. Gastrointest. Liver Physiol. 246, G204–G208 6141741 10.1152/ajpgi.1984.246.2.G204

[44] Cheng Y., and Prusoff W. H. (1973) Relationship between the inhibition constant (*K_I_*) and the concentration of inhibitor which causes 50 per cent inhibition (*I*_50_) of an enzymatic reaction. Biochem. Pharmacol. 22, 3099–3108 10.1016/0006-2952(73)90196-2 4202581

